# Supplementary material for: ZMYND10 functions in a chaperone relay during axonemal dynein assembly
Source: eLife. 2018 Jun 19;7:e34389. doi: 10.7554/eLife.34389 (PMC6044906; doi:10.7554/eLife.34389)
Supplement: Source data 1. — Images show un-cropped versions of immunoblots. Figure panels used are demarcated with a box and the detected proteins are labeled. Asterisks are used to denote either non-specific bands (6C, 6D and 7B) or putative degradation products (7E). HE = High exposure, LE = Low exposure [file elife-34389-data1.pdf]

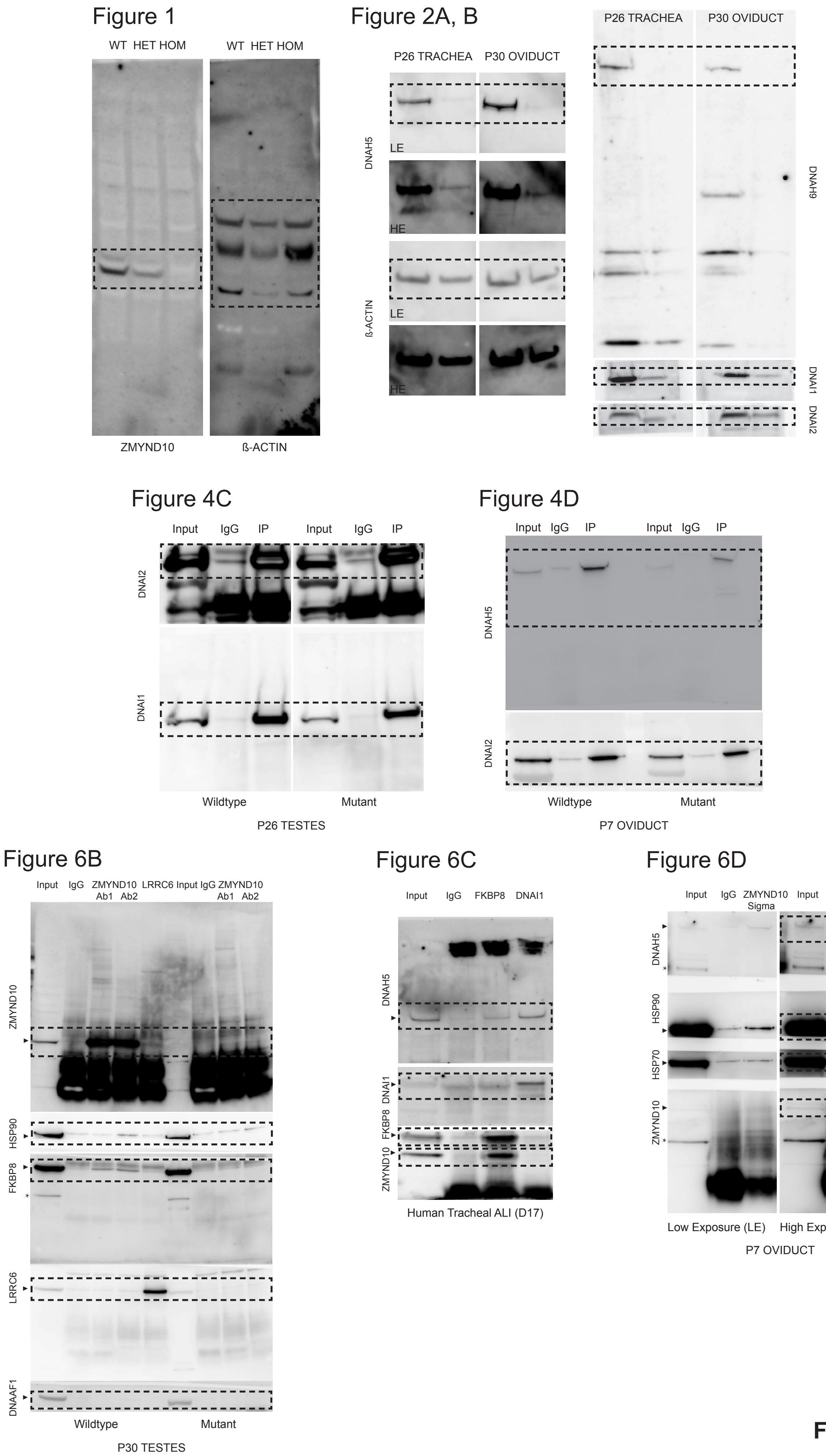

Figure 1
WT HET HOM
WT HET HOM
ZMYND10
ß-ACTIN
Figure 2A, B
P26 TRACHEA
P30 OVIDUCT
DNAH5
LE
HE
ß-ACTIN
LE
HE
P26 TRACHEA
P30 OVIDUCT
DNAH9
DNAI1
DNAI2
Figure 4C
Input
IgG
IP
Input
IgG
IP
DNAI2
DNAI1
Wildtype
Mutant
P26 TESTES
Figure 4D
Input
IgG
IP
Input
IgG
IP
DNAH5
DNAI2
Wildtype
Mutant
P7 OVIDUCT
Figure 6B
Input
IgG
ZMYND10
Ab1
Ab2
LRRC6
Input
IgG
ZMYND10
Ab1
Ab2
ZMYND10
HSP90
FKBP8
LRRC6
DNAAF1
Wildtype
Mutant
P30 TESTES
Figure 6C
Input
IgG
FKBP8
DNAI1
DNAH5
DNAI1
FKBP8
ZMYND10
Human Tracheal ALI (D17)
Figure 6D
Input
IgG
ZMYND10
Sigma
Input
IgG
ZMYND10
Sigma
DNAH5
HSP90
HSP70
ZMYND10
Low Exposure (LE)
High Exposure (HE)
P7 OVIDUCT

Figure S6.

Figure 7B

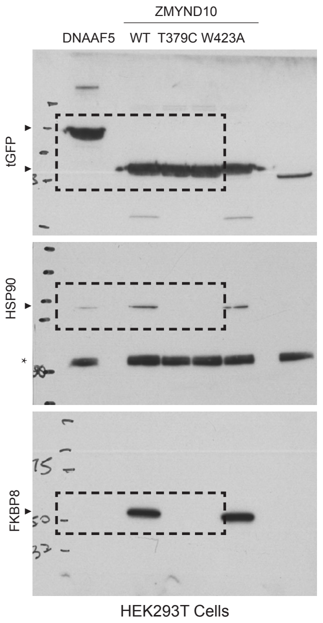

Figure 7C

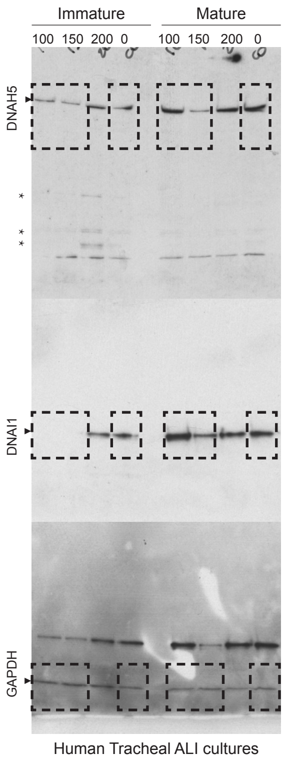

Figure 8A

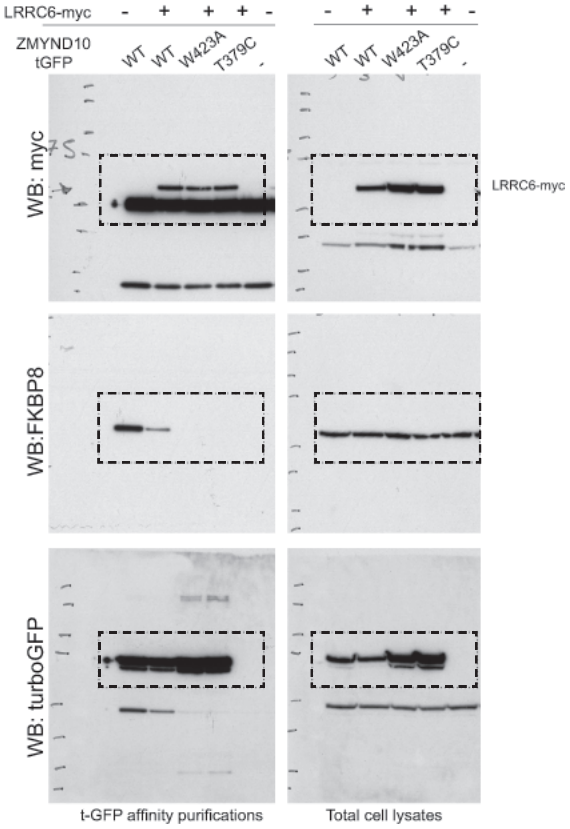

Figure S4B, C

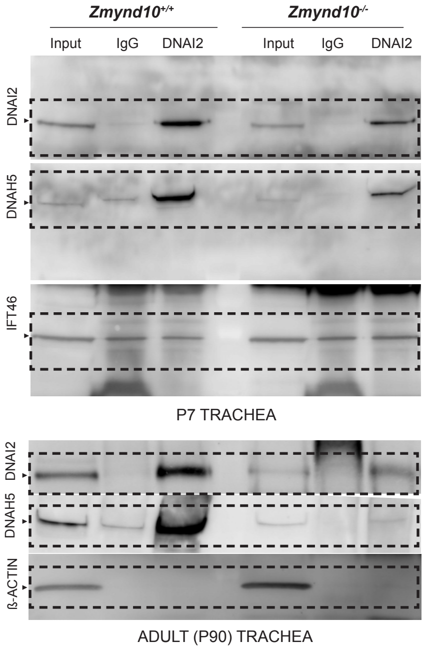

Figure S6 continued.
