# Supplementary material for: ZMYND10 functions in a chaperone relay during axonemal dynein assembly
Source: eLife. 2018 Jun 19;7:e34389. doi: 10.7554/eLife.34389 (PMC6044906; doi:10.7554/eLife.34389)
Supplement: Supplementary file 4. [file elife-34389-supp4.pdf]

Gene (mouse)	Forward (5'-3')	Reverse (5'-3')	Usage description
Zmynd10	CTGATTGCGGTGGAGATGTG	CTTCTCCAGGCTCTCATGCT	LEFT_PRIMER 223 RIGHT_PRIMER 123
	GCATTTTGAGAGTGGCCGAT	TGCTCAGGGAAAGGCTATCC	LEFT_PRIMER 711 RIGHT_PRIMER 613
	TATTGCTGCAGGGAGTGTCA	AGAGGCTGAATACGTGCTGA	LEFT_PRIMER 1237 RIGHT_PRIMER 1090
Zmynd10	ACTCAAGCGCTTCAAC	CAAGCCCCAGAATACCTT	*Zmynd10* cDNA RT-PCR (Exons 1-3)
	CATATACATGGTGGTAC	AGCTGCAAAAGCAGGCGGA	*Zmynd10* cDNA RT-PCR (Exons 3/4-6)
	ATGGAATTTGAGATATCCCT	AGCAAAAGCTGAATAAACTG	*Zmynd10* cDNA RT-PCR (Exons 6-8)
	ATGGGCAAGTATGGATCGCCCT	AACAGATCCCAGAAATCTGGGAT	*Zmynd10* cDNA RT-PCR (Exons 8-9)
	TGGAGAGAGAGAACAAAGGGAAAT	TGGTATTGCTGCAGGGAGTGT	*Zmynd10* cDNA RT-PCR (Exons 10-12)
	AGTCAAGCACTGGGAGAAGCAC	TTGGGGTCGGGGGAGAAGA	*Zmynd10* cDNA RT-PCR (Exon12-UTR)
Zmynd10	TCAAGTCAAGCACGCGGAGAAGCACGG	TCCGTGCTTCTCCGCGTGCTTGACTTG	Mutagenesis W432A
	AGGTGGGCTGAATGCTACAGGCTGG	TCCAGCCTGTAGCATTCAGCCCACC	Mutagenesis T379C
	GGACTTTCCAAAATGTCG	GGTAGGTGCCGAAGTGGTAG	Seq pCMV6-AC-tGFP
	CCTAAGAAGGACCTAGTGTTAG	CGGGCCACTAGCAGTATCAG	Sequence Validation ORF Zmynd10
	TCCCTCGTTTCCATGGCGG	AGATTGGGCAACTGGTCGAG	Sequence Validation ORF Zmynd10
	CATGGGAAGATCCCAACGCT	TGTCTTTCCGTGCTTCTCCC	Sequence Validation ORF Zmynd10
	TCTCGGTGCTTCGCTACATC		Sequence Validation ORF Zmynd10
