# Supplementary material for: ZMYND10 functions in a chaperone relay during axonemal dynein assembly
Source: eLife. 2018 Jun 19;7:e34389. doi: 10.7554/eLife.34389 (PMC6044906; doi:10.7554/eLife.34389)
Supplement: Transparent reporting form [file elife-34389-transrepform.pdf]

eLIFE
1st Floor
24 Hills Road
Cambridge CB2 1JP, UK
P 01223 855340
W elifesciences.org
T @elife

eLife's transparent reporting form

We encourage authors to provide detailed information *within their submission* to facilitate the interpretation and replication of experiments. Authors can upload supporting documentation to indicate the use of appropriate reporting guidelines for health-related research (see EQUATOR Network), life science research (see the BioSharing Information Resource), or the ARRIVE guidelines for reporting work involving animal research. Where applicable, authors should refer to any relevant reporting standards documents in this form.

If you have any questions, please consult our Journal Policies and/or contact us: editorial@elifesciences.org.

Sample-size estimation

- You should state whether an appropriate sample size was computed when the study was being designed
- You should state the statistical method of sample size computation and any required assumptions
- If no explicit power analysis was used, you should describe how you decided what sample (replicate) size (number) to use

Please outline where this information can be found within the submission (e.g., sections or figure legends), or explain why this information doesn't apply to your submission:

For our immunofluorescence or imaging based studies (**Figures 1C, 1D, 1F, 2C, 2D, 3, 4A, 4B**, **5A,5B, see also Figure 1- figure supplements 2D, 3B and Figure 4- figure supplement 4A**) we compare the proportion with a dichotomous outcome (i.e. stability/destabilized, motility/immotile) between 2 samples (control versus mutant), using a Chi-squared statistic (or z test) assuming a 5% level of significance; power of the study of 80%, p1= 0.01, p2=0.99 predicts our standardized effect size should be ~3 animals (biological replicates) per group. Where possible, we used sex matched littermates as contorls (i.e. wild type of heterozygote animals). Representative images were selected for publication. This information is outlined in the Materials and Methods section.

A similar strategy was used for our label-free quantitative proteomics (**Figure 5D**), where 3 sets of P25 testes (biological replicates) per group were isolated and run. This information is outlined in the Materials and Methods section.

Replicates

- You should report how often each experiment was performed
- You should include a definition of biological versus technical replication
- The data obtained should be provided and sufficient information should be provided to indicate the number of independent biological and/or technical replicates
- If you encountered any outliers, you should describe how these were handled
- Criteria for exclusion/inclusion of data should be clearly stated
- High-throughput sequence data should be uploaded before submission, with a private link for reviewers provided (these are available from both GEO and ArrayExpress)

eLife Sciences Publications, Ltd is a limited liability non-profit non-stock corporation incorporated in the State of Delaware, USA, with company number 5030732, and is registered in the UK with company number FC030576 and branch number BR015634 at the address 1st Floor, 24 Hills Road, Cambridge CB2 1JP | August 2014

Please outline where this information can be found within the submission (e.g., sections or figure legends), or explain why this information doesn't apply to your submission:

For our gross mouse phenotyping (**Figure 1, Figure 1- supplement 2**), we analyzed more than 3 animals per genotype for stage (biological replicates). Representative images are shown in the figures. For the molecular and cellular characterization (**Figures 1B, 2,3,4, 5A,B**), motile ciliated tissues were dissected or isolated from at least 3 animals per genotype per stage (biological replicates), with littermate controls (either wild type or heterozygote) where possible. In addition, technical triplicate repeats were done for qRT-PCR (**Figure 2E**).

For the interaction studies (**Figures 4C-F, 6A,6B**) comparing wild type to mutant extracts, we analyzed at least 3 animals per genotype (biological replicates), although density quantification (**Figure 4E**) represents the pictured (most representative) immunoblot only for clarity. For interaction studies pooled extracts from mouse testes (P30, N=3 wild type animals **Figure 6A,6B**) or oviduct (P7, N=3 wild type animals **Figure 6D**) were used. For our interaction studies with turboGFP, experiments were repeated with transient transfections 3 times (**Figure 7B,8A**). Finally, for our analysis of heavy chain interactions (**Figure 6C**), we used extracts from a single human donor MucilAir immature (D17), using 3 inserts per IP to minimize variation across inserts. Individual immunoprecipitations were repeated twice with these extracts (technical repeats).

For our small molecule inhibitor studies (**Figure 7C,D**), we used MucilAir inserts from two different human donors (batch number MD058002 and MD068001), differentiated to D60 (mature) and D17 (immature). Experiments were repeated twice with varying concentrations (biological replicates, N) and extracts from these samples were run twice (technical replicates, n).

eLife Sciences Publications, Ltd is a limited liability non-profit non-stock corporation incorporated in the State of Delaware, USA, with company number 5030732, and is registered in the UK with company number FC030576 and branch number BR015634 at the address 1st Floor, 24 Hills Road, Cambridge CB2 1JP | August 2014

Statistical reporting

- Statistical analysis methods should be described and justified
- Raw data should be presented in figures whenever informative to do so (typically when N per group is less than 10)
- For each experiment, you should identify the statistical tests used, exact values of N, definitions of center, methods of multiple test correction, and dispersion and precision measures (e.g., mean, median, SD, SEM, confidence intervals; and, for the major substantive results, a measure of effect size (e.g., Pearson's r, Cohen's d)
- Report exact p-values wherever possible alongside the summary statistics and 95% confidence intervals. These should be reported for all key questions and not only when the p-value is less than 0.05.

Please outline where this information can be found within the submission (e.g., sections or figure legends), or explain why this information doesn't apply to your submission:

For statistical analysis of dynein gene expression by qRT-PCR (**Figure 2E**) we extracted RNA from oviducts of three female mice per genotype (control versus mutants) and performed qRT-PCR runs in triplicate (N=3, n=3). The graph shows relative gene expression with error bars representing standard error of the mean. Significance of gene expression differences between controls and mutants was tested using a paired two tailed t-test. For *Dnah5* the t-test p-value was 0.28 and for *Dnali1* it was 0.79 indicated as not significant (**n.s. in Figure 2E**). This information can be found in the Materials and Methods section.

To compare proteome wide changes between control and mutants, we used age matched testes samples. This information is outlined in the Materials and Methods "Mass Spectrometry and proteomic data analysis". Three biological replicates per genotype were used and three technical runs were performed on the Mass Spectrometer (N=3, n=3). Fold changes were calculated (Mean/Mean), centre was set to Mean and t-test statistics computed. Permuation-based FDR multiple test correction was used. This data is summarised in **Figure 5D** and p-values listed in **Supplementary file 1**.

For analysis of endogenous ZMYND10 IP mass spectrometry data (See Materials and Methods), we used a student's t-test to compare fold changes between control pulldowns and ZMYND10 pulldowns. We assumed normal distribution of the intensity values. In addition we applied a ratio cut-off (Mean/Mean), centre was set to Mean. Samples were run in duplicate using one pooled biological replicate (N=1, n=2) but using two separate ZMYND10 polyclonal antibodies. Common hits are highlighted in a Venn diagram in **Figure 6A** and p-value statistics are listed as -log 10 transformed values in **Supplementary file 2.**

(For large datasets, or papers with a very large number of statistical tests, you may upload a single table file with tests, Ns, etc., with reference to sections in the manuscript.)

Group allocation

- Indicate how samples were allocated into experimental groups (in the case of clinical studies, please specify allocation to treatment method); if randomization was used, please also state if restricted randomization was applied

eLife Sciences Publications, Ltd is a limited liability non-profit non-stock corporation incorporated in the State of Delaware, USA, with company number 5030732, and is registered in the UK with company number FC030576 and branch number BR015634 at the address 1st Floor, 24 Hills Road, Cambridge CB2 1JP | August 2014

- Indicate if masking was used during group allocation, data collection and/or data analysis

Please outline where this information can be found within the submission (e.g., sections or figure legends), or explain why this information doesn't apply to your submission:

For most of our studies, randomization is not applicable but blinding is used, such that mouse genotypes are not displayed on cage cards. Similarly, gross phenotyping (**Figure 1** and **Figure 1 - supplement 2**) and proteomic profiling (**Figure 5D**) are performed without knowledge of the mouse genotype where possible. We will also use blinding to avoid scoring bias in our imaging experiments by using randomised fields of view, where possible, followed by recoding of image filenames by computer script prior to analysis.

Additional data files ("source data")

- We encourage you to upload relevant additional data files, such as numerical data that are represented as a graph in a figure, or as a summary table
- Where provided, these should be in the most useful format, and they can be uploaded as "Source data" files linked to a main figure or table
- Include model definition files including the full list of parameters used
- Include code used for data analysis (e.g., R, MatLab)
- Avoid stating that data files are "available upon request"

Please indicate the figures or tables for which source data files have been provided:

The complete proteomic dataset whose summary is graphically represented in **Figure 5D** is available via ProteomeXchange with identifier PXD006849 and is summarized in **Supplementary file 1**.
Testes Whole Proteome Dataset (PRIDE database):
Project Name: ZMYND10 functions in a chaperoneh relay during axonemal dynein assembly
Project accession: PXD006849
Reviewer account details:
Username: reviewer18571@ebi.ac.uk
Password: oWarXaXa

The ZMYND10 endogenous IP interaction dataset graphically summarized in **Figure 6A** is available via ProteomeXchange with identifier PXD006849 and is summarized in **Supplementary File 2**.

eLife Sciences Publications, Ltd is a limited liability non-profit non-stock corporation incorporated in the State of Delaware, USA, with company number 5030732, and is registered in the UK with company number FC030576 and branch number BR015634 at the address 1st Floor, 24 Hills Road, Cambridge CB2 1JP | August 2014
